# Randomized Phase 2 Study Comparing Pathological Responses of Resected Colorectal Cancer Metastases after Bevacizumab with mFOLFOX6 or FOLFIRI (BEV-ONCO Trial)

**DOI:** 10.3390/cancers14051183

**Published:** 2022-02-24

**Authors:** Pamela Baldin, Javier Carrasco, Gabriela Beniuga, Anne Jouret-Mourin, Gauthier Demolin, Sandrine Roland, Lionel D’Hondt, Philippe Vergauwe, Daniel Van Daele, Marie Mailleux, Isabelle Sinapi, Astrid De Cuyper, Noëlla Blétard, Brigitte Massart, Monique Delos, Marie-Laure Castella, Aline van Maanen, Marc Van den Eynde

**Affiliations:** 1 Pathology Department, Cliniques Universitaires Saint Luc (UCL)—Université Catholique de Louvain, 1200 Bruxelles, Belgium; pamela.baldin@uclouvain.be (P.B.); anne.mourin@uclouvain.be (A.J.-M.); 2 Department of Medical Oncology, GHdC-Grad Hopital de Charleroi-Site Notre Dame, 6000 Charleroi, Belgium; javier.carrasco@ghdc.be (J.C.); isabelle.sinapi@ghdc.be (I.S.); 3 Pathology Department, Institut de Pathologie et Génétique, 6041 Gosselies, Belgium; gabriela.beniuga@ipg.be; 4 Gastroenterology Department, Clinique CHC MonLégia, 4000 Liège, Belgium; gauthier.demolin@chc.be; 5 Gastroenterology Department, CHIREC-Hôpital Delta, 1160 Auderghem, Belgium; sandrine.roland@chirec.be; 6 Oncology Department, CHU-UCL-Namur, Site Godinne, 5530 Yvoir, Belgium; lionel.dhondt@uclouvain.be; 7 Gastroenterology Department, AZ Groeninge Hospital, 3220 Kortrijk, Belgium; philippe.vergauwe@azgroeninge.be; 8 Gastroenterology Department, CHU de Liège, 4000 Liège, Belgium; daniel.vandaele@chu.ulg.ac.be; 9 Medical Oncology, Clinique Saint-Luc Bouge, 5000 Namur, Belgium; marie.mailleux@slbo.be; 10 Department of Medical Oncology, Cliniques Universitaires Saint Luc (UCL)—Université Catholique de Louvain, 1200 Bruxelles, Belgium; astrid.decuyper@uclouvain.be; 11 Pathology Department, Clinique CHC MonLégia, 4000 Liège, Belgium; noella.bletard@chc.be (N.B.); brigitte.massart@chc.be (B.M.); 12 Pathology Department, CHU-UCL-Namur, Site Godinne, 5530 Yvoir, Belgium; monique.delos@uclouvain.be; 13 Colorectal Clinical Research Unit, Institut Roi Albert II, Cliniques Universitaires Saint Luc (UCL)—Université Catholique de Louvain, 1200 Bruxelles, Belgium; marie-laure.castella@uclouvain.be; 14 Support Statistique, Institut Roi Albert II, Cliniques Universitaires Saint Luc (UCL)—Université Catholique de Louvain, 1200 Bruxelles, Belgium; aline.vanmaanen@uclouvain.be; 15 Department of Medical Oncology and Gastroenterology, Cliniques Universitaires Saint Luc (UCL)—Université Catholique de Louvain, 1200 Bruxelles, Belgium

**Keywords:** bevacizumab, chemotherapy, colorectal liver metastases, pathological response, histological growth pattern, tumoral homogeneity

## Abstract

**Simple Summary:**

Nowadays, the surgery of liver metastases remains the only hope of a cure for patients with colorectal cancer. Pathological responses evaluated after preoperative treatment strongly influences the risk of relapse and patient survival. Previous studies reported that preoperative bevacizumab combined with an oxaliplatin-based chemotherapy provided a higher pathological response rate compared with an irinotecan-based regimen or chemotherapy alone. This prospective trial, having recruited 65 patients with resectable colorectal liver metastases, ambitioned to report a higher major pathological response rate after mFOLFOX6-bevacizumab compared to FOLFIRI-bevacizumab. Among the 57 patients with 159 resected metastases, no difference in major pathological response rate was observed between treatments. Nevertheless, the trial prospectively confirmed the pathological response of resected colorectal liver metastases as a significant biomarker for tumor recurrence, justifying its implementation in clinical practice. Interestingly, we observed that the homogeneity of the pathological response and histological growth pattern of liver metastases was also strongly associated with patient’s survival.

**Abstract:**

Retrospective studies reported that preoperative oxaliplatin-based chemotherapy increased pathological response (PR) in patients resected for colorectal liver metastases (CRLM). This multicenter prospective randomized (1/1) phase II trial evaluated PR on resected CRLM after preoperative mFOLFOX6 (arm A) or FOLFIRI (arm B) + bevacizumab. The primary endpoint was the major pathological response rate (MPRR), defined as the percentage of patients presenting CRLMs with mean tumor regression grade (TRG) < 3. Secondary endpoints included safety, progression-free survival (PFS) and overall survival (OS). Out of 65 patients, 57 patients (28 and 29 in arm A/B) were resected for CRLM (one patient with lung metastases). Clinical and treatment characteristics were similar in both arms. One-month postoperative complications were 39.3%/31.0% in arm A/B (*p* = 0.585). MPRR and complete PR were 32.1%/20.7% (*p* = 0.379) and 14.3%/0.0% (*p* = 0.052) in arm A/B, respectively. PFS and OS were not different. Patients with PR among all CRLMs (max TRG ≤ 3; 43.8% of patients) had a lower risk of relapse (PFS: HR = 0.41, 95%CI = 0.204–0.840, *p* = 0.015) and a tendency towards better survival (OS: HR = 0.34, 95%CI = 0.104–1.114, *p* = 0.075). The homogeneity of PR was associated with improved PFS/OS. This trial fails to demonstrate a significant increase in MPRR in patients treated with mFOLFOX6-bevacizumab but confirms PR as an important prognostic factor.

## 1. Introduction

Colorectal cancer is the third most common cancer in the world with an increasing incidence, especially in younger adults [1]. Studies showed that up to 50% of patients develop colorectal liver metastases (CRLM) in the course of the disease [2], and the majority of them will die due to this involvement. Chemotherapy combined with biological therapies was shown to improve overall survival in metastatic CRC (mCRC) and increase the number of patients candidate for resection [3].

Few prospective trials assessed the role of chemotherapy with or without targeted therapies for resectable CRLM. The EPOC study evaluating peri-operative FOLFOX chemotherapy reported improved disease-free survival (DFS) but failed to demonstrate long-term overall survival (OS) benefits compared to patients treated with surgery only [4]. More recently, the addition of cetuximab (anti-EGFR monoclonal antibody improving OS in inoperable mCRC) to FOLFOX, for patients with *KRAS* wild-type mCRC, conferred significant DFS and OS disadvantages compared to perioperative FOLFOX only [5]. These results contrasted with the previous CELIM trial reporting a higher tumor response rate and increased resectability when cetuximab was combined with FOLFOX or FOLFIRI for unresectable CRLM [6]. Several trials investigated the role of bevacizumab, an anti-VEGF monoclonal antibody, combined with chemotherapy for potentially or borderline resectable CRLM. These small non-randomized and controlled phase 2 studies reported interesting responses and liver resection rates [7,8,9]. Even if it currently remains unclear whether chemotherapy should be administered before metastatic resection, commonly, 5-fluorouracil/leucovorin/oxaliplatin (FOLFOX), or less frequently, 5-fluorouracil/leucovorin/irinotecan (FOLFIRI), are used.

Several studies reported the prognostic survival relevance of some clinico-pathological parameters after CRLM surgery, such as size and the number of lesions [10], status of the surgical margin [11], pathological response (PR) assessed by tumor regression grading (TRG) [12,13,14,15], histopathological growth pattern (HGP) of liver metastases [16,17], molecular status assessed by the presence of *RAS* and *BRAF* mutations [3,18], chemotherapy-associated liver injury (CALI) [19,20] and Immunoscore [20,21,22]. We reported recently that a complete pathological evaluation of metastasis and surrounding liver parenchyma permitted the adequate stratification of resected mCRC patient prognosis [20]. The presence of steatohepatitis, replacement or mixed HGP, more than three CRLM and positive surgical margin (R1) were associated with a higher risk of tumor recurrence.

TRG is an important prognostic factor in patients resected for CRLM. A retrospective study from Rubbia-Brant et al. [12] showed that PR in resected CRLM allowed for the efficacy of chemotherapy to be evaluated and was correlated with prognosis and survival. Patients presenting a major pathological response rate (MPRR) (TRG < 3) had an improved 3-year DFS and 5-year OS compared with patients with no PR (TRG 4–5). Other retrospective studies [13,14] or meta-analysis [15] reported that patients treated with preoperative FOLFOX-Bevacizumab had a higher rate of MPRR compared to those with preoperative FOLFIRI-Bevacizumab treatment or chemotherapy alone.

The aim of the BEV-ONCO trial is to evaluate, in a randomized prospective setting, the rate of MPRR in resected CRLM after a preoperative treatment with mFOLFOX6/bevacizumab or FOLFIRI/bevacizumab.

## 2. Materials and Methods

### 2.1. Study Design and Patients

BEV-ONCO (NCT01858649) is a prospective, randomized, Belgian, multicenter phase II study (Figure S1, see Supplementary Materials and Methods) including mCRC patients with resectable CRLM, for which the decision of preoperative chemotherapy was considered in a multidisciplinary meeting. Additional key inclusion criteria required were: age ≥ 18 years-old; EGOG performance status ≤ 1; adequate hematological, renal and hepatic functions; and no previous systemic therapy for mCRC. Adjuvant oxaliplatin-based chemotherapy completed at least 1 year before trial inclusion and with peripheric neuropathy < grade 2 was allowed. Included patients were randomized (1/1) and treated with a minimum of 3 to maximum of 6 cycles of preoperative chemotherapy: mFOLFOX6-bevacizumab for arm A and FOLFIRI-bevacizumab for arm B. Bevacizumab was interrupted at least 6 weeks before surgery (the last preoperative cycle of chemotherapy could be given without bevacizumab). Surgical resection of CRLM was performed within 4 to 8 weeks after the last chemotherapy cycle according to local procedure. Postoperative treatment was administrated according to investigator decision (optional).

The trial was approved by institutional ethical committees at all participating centers. The trial conformed to the principles outlined in the Declaration of Helsinki and was conducted in accordance with the EU Directive 2001/20/EC and the Good Clinical Practice for Trials of Medical Products in the European Community. Written informed consent was provided by participants.

### 2.2. Pathological Evaluation

A similar methodology for the sampling of resected CRLM was required across all the participating centers (see Supplementary Materials and Methods). All CRLM were sampled for analysis, in toto where possible. Additionally, samples from surrounding liver parenchyma were collected. Samples were formalin-fixed and paraffine-embedded (FFPE), cut in 5 μm thick sections and examined microscopically. Morphological analysis was centrally reviewed by 3 expert pathologists (PB, GB, AJM), using H&E, Masson’s trichrome blue and reticulin staining, and the histological diagnosis was made according to WHO 2019 criteria [23]. The pathological response of each metastasis was scored according to TRG classification [12]. TRG is a semi-quantitative classification system comprising of 5 grades (TRG 1–5) based on the proportion of tumoral cells and fibrosis in the tumor (Figure S2A). High TRG (TRG 4–5) reflects non pathological response and low TRG (TRG 1–2–3) reflects complete, major or minor pathological response. In patients with multiple CRLM, TRG is assessed as max-TRG (the higher TRG among all the lesions), mean TRG (the mean of all TRG), homogeneous TRG (when all the CRLM of the patient had the same TRG) and low homogeneous TRG (when all CRLMs of the patient had the same TRG and lower than 3).

HGP was assessed based on the morphology of the tumor–non-tumor–liver interface, as described by Eefsen et al. [24]: desmoplastic HGP, pushing HGP, replacement HGP and mixed HGP (Figure S2B). Mixed HGP corresponded to tumors comprising more than 1 pattern in the same lesion. For patients with several metastases, HGP was assessed as replacement and mixed HGP (when all the lesions of the patient presented replacement and/or mixed HGP), HGP-dominant desmoplastic (when the majority of the CRLM per patient presented a desmoplastic pattern) and homogeneous HGP (when all the lesions of the patient presented the same pattern with the exception of mixed patterns that were considered heterogeneous by definition). In the nontumoral hepatic parenchyma, CALI, including sinusoidal obstruction syndrome (SOS), nodular regenerative hyperplasia (NRH) and steatohepatitis (Figure S2C), was assessed as previously described [20,25,26,27].

A positive resection margin (R1 status) was defined when the lesion crossed the surgical margin. In cases of multiple metastases, the resection margin was assessed as positive if at least 1 lesion was positive.

Finally, we assessed a pathological score as we previously reported [20]. Pathological score was calculated by adding 1 point when one of the following criteria were present: more than 3 lesions, R1-positive margin, replacement or mixed HGP and steatohepatitis.

### 2.3. Objectives, Statistical Considerations and Analyses

The primary endpoint was MPRR, defined as the percentage of patients presenting with CRLM with a mean TRG lower than 3. Secondary endpoints included patient’s safety (preoperative toxicity and one-month surgical complication rate), progression-free survival (PFS) and OS (see Supplementary Materials and Methods). Other pathological objectives included: complete PR, complete resection rate, presence of CALI such as SOS, NRH and steatohepatitis. The significance of HGP and homogeneity of tumor response was also further investigated.

A sample size of 54 patients (27 per arm) was needed to achieve 80% power to detect a difference between the group proportions of 0.40 for MPPR. The proportion of MPPR in the treatment group FOLFIRI + bevacizumab was assumed to be 0.20. Type I error was set as 0.05. With an expected drop-out rate of 10%, 60 subjects were randomized. Continuous and categorical variables were analyzed with the Mann-Whitney U-test and two-sided Fisher’s Exact test, respectively. PFS and OS were summarized using Kaplan-Meier curves. Univariate logistic regression or Cox proportional hazard modelling was used appropriately to identify factors affecting pathological variable (TRG, HGP) or survival risk factors. Backward stepwise selection was used to select optimal multivariate models for OS and PFS. Potential collinearity was tested among the multiple parameters, significantly associated with survival using the variance indicator factor (VIF) and the collinearity indices (COLLIN). Analysis was performed using SAS software (Version 9.4; SAS Institute Inc., Cary, NC, USA). *p*-values of less than 0.05 were considered statistically significant.

## 3. Results

### 3.1. Patients

**Figure 1:**
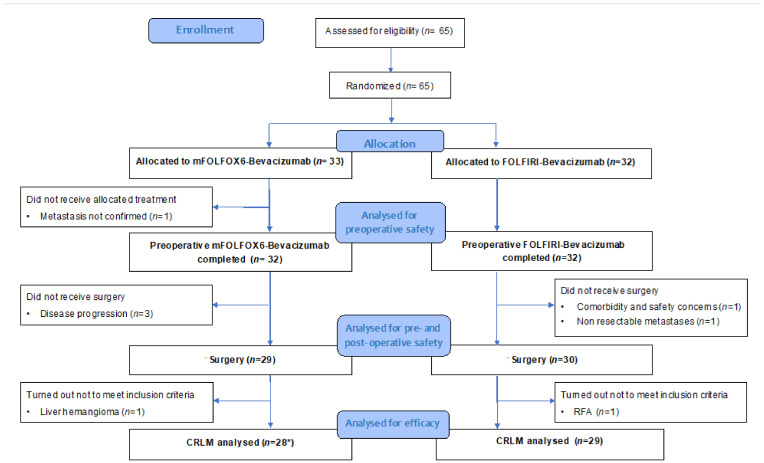
CONSORT flow diagram of the trial. CRLM: colorectal liver metastasis; RFA: radiofrequency ablation. * Including 1 patient with lung metastases.

Between June 2013 and September 2018, 65 patients were randomized in the BEV-ONCO trial (Table S1). Thirty-three patients were assigned to arm A (mFOLFOX6-Bev) and 32 to arm B (FOLFIRI-Bev), of which 28 (84.8%) and 29 (90.6%) were resected for CRLM, respectively (Figure 1).

The baseline clinical characteristics were not different between the two arms (Table 1). Overall, the median age was 60 years old, 51% of patients were male, 33% *RAS* wild-type, one patient presented lung metastases, 75% of CRLM cases were synchronous and patients received a median of four chemo cycles and three bevacizumab cycles preoperatively. Sixteen patients (28.1%) underwent major hepatectomy requiring preoperative portal vein embolization. Two steps hepatectomy was performed for four patients.

### 3.2. Safety

Preoperative and one-month postoperative complications were similar in the two arms of the study (Table 2). Out of 64 patients receiving preoperative treatment, five (15.6%) and seven (21.9%) presented grade 3–4 adverse events in arm A/B, respectively (*p* = 0.750). Nineteen patients (29.7%) presented adverse events of special interest (related to treatment or disease evolution, listed in Table 2), which were not different between arms.

Out of 57 patients, one-month postoperative complications occurred in 20 patients (35.1%). No differences in terms of frequency and gradation of adverse events were observed between treatments arms. Grade 3–4 postoperative complications, mainly including cardio-vascular events, surgery leakage and intra-abdominal/wound infections, were not different regarding the treatment arm (arm A: 17.9%, arm B: 6.9%, *p* = NS).

### 3.3. Pathological Results

In total, 159 CRLM were resected and evaluated, 89 in arm A and 70 in arm B (Table S2).

General pathological characteristics were similar in the two arms concerning the number of resected lesions (median of 2 mm per patients), the size (median 15 mm) and the completeness of resection (R0 resection in arm A/B: 89.3%/93.1%) (Table 1). MPRR (mean TRG < 3) was 32.1% in arm A and 20.7% in arm B (*p* = 0.379).

Four patients presented complete PR in arm A (14.3%) and none in arm B (*p* = 0.052). The proportion of patients with a max-TRG ≤ 3 was similar between both arms (50% vs. 37.9%; *p* = 0.429). No difference between arms was observed for SOS (arm A/B: 53.6%/37.9%, *p* = 0.501), NRH (arm A/B: 21.4%/17.2%, *p* = 0.752), steatohepatitis (arm A/B: 10.7%/13.8%, *p* = 0.999) and pathological score (>1, arm A/B: 25.0%/17.2%, *p* = 0.530).

The clinical and pathological characteristics of patients with a pathological response in resected CRLM (max TRG ≤ 3) are reported in Table 3.

PR was associated with clinical factors, including metachronous CRLM (*p* = 0.022), the presence of one lesion (*p* = 0.005), a median size < 20 mm (*p* = 0.014), and less than three preoperative administered cycles of bevacizumab (*p* = 0.005) and chemotherapy (*p* = 0.042). The pathological parameters significantly associated with PR are absence of replacement HGP and mixed HGP (*p* = 0.007) and the presence of a HGP dominant desmoplastic pattern (*p* = 0.011). We performed additional exploratory analyses regarding histological patterns associated with response. Interestingly, patients (with single or multiple CRLM) presenting a pathologic homogenous evolution after systemic treatment with a homogenous TRG and HGP among all their CRLM had a greater association with pathological response (*p* < 0.001 and *p* = 0.050, respectively). Pathological score and CALI (SOS, NRH, steatohepatitis) were not associated with a TRG ≤ 3.

### 3.4. Survival Outcome

**Figure 2:**
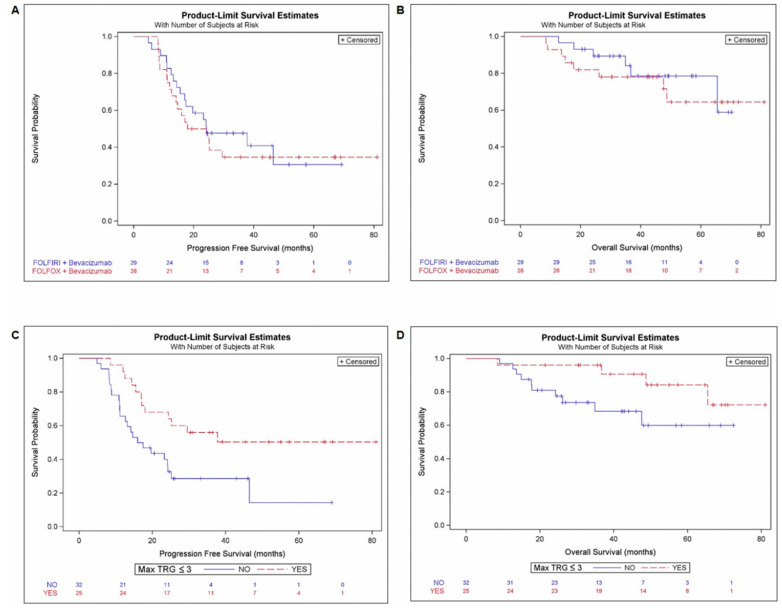
PFS and OS according treatment arm and max TRG ≤ 3. Kaplan-Meier curves for PFS (**A**) and OS (**B**) according to the treatment arm (mFOLFOX6-bevacizumab and FOLFIRI-bevacizumab). Kaplan-Meier curves for PFS (**C**) and OS (**D**) according to pathological response reflecting by MaxTRG ≤ 3 (yes/no).

There was no survival significant difference depending on the type of treatment. No difference was observed for PFS (arm A/B: HR: 1.18, 95%CI: 0.607–2.291, *p* = 0.626) and OS (arm A/B: 1.38, 95%CI: 0.480–4.000, *p* = 0.550) (Table 4, Figure 2A,B).

Independently of the treatment arm, the 25 (43%) patients with a max TRG ≤ 3 among resected CRLM (Max TRG ≤ 3; 43.8% of pts) had a significantly lower risk of relapse (PFS: HR = 0.41, 95%CI = 0.202–0.835, *p* = 0.014) and tended to have a better survival (OS: HR = 0.34, 95%CI = 0.105–1.114, *p* = 0.075) (Table 4; Figure 2D).

Additionally, metachronous metastases, the presence of one lesion, negative surgical margin absence of replacement and mixed HGP and a pathological score lower or equal to 1 were significantly associated with longer PFS.

Interestingly, in our exploratory analyses, the homogeneity of TRG and HGP after systemic treatment seems to be significantly associated with survival outcome (Table 4 and Figure S3). The presence of homogeneous TRG, low homogeneous TRG and homogeneous HGP was associated with a significant longer PFS. Longer OS was associated with left tumor sidedness but also with homogeneous TRG and HGP. CALI was not associated with prognosis in the univariate analysis.

### 3.5. Homogeneity of Pathological Response and Histological Growth Pattern

Regarding the association with pathological response and patient’s outcome, we further explored the parameters associated with CRLM homogeneity. The univariate logistic regression for homogeneous TRG (Table S3) demonstrated a significant association with less than three preoperative cycles of bevacizumab (*p* = 0.016), chemotherapy (*p* = 0.036), and homogeneous HGP (*p* = 0.004). Interestingly, the presence of SOS was inversely associated with a homogenous TRG (*p* = 0.050).

Univariate logistic regression for homogeneous HGP (Table S3), reported a significant association with metachronous disease (*p* = 0.037), homogeneous TRG (*p* = 0.012), low homogeneous TRG (*p* = 0.002), an absence of replacement HGP and mixed HGP (*p* < 0.001), HGP-dominant desmoplastic (*p* = 0.003), a pathological score lower or equal than 1 (*p* < 0.001) and absence of a SOS pattern (*p* = 0.048).

After checking that there was no collinearity (Table S4) between the parameters significantly associated with PFS and OS (Table 4) and with max TRG ≤ 3 (Table 3) in the univariate analyses (lesion number, synchronous metastases, homogeneous TRG and HGP), a multivariate analysis was performed. Despite a significant association with max TRG ≤ 3, only homogenous TRG remained significantly associated with both OS and PFS in the multivariate analysis; homogenous HGP was only significant for PFS but not for OS (Table S5).

To support the possible relevance of the homogeneity of pathological parameters and exclude the bias of patients with one single lesion, we conducted additional analyses comparing patients with one lesion and multiple CRLM (Table S6). Eighteen (51.4%) out of thirty-five patients with multiple lesions presented homogeneous TRG. Homogeneous HGP was observed in 19 (86.4%) patients with 1 lesion and in 34 (59.6%) patients with multiple lesions.

Clinico-pathologic parameters associated with homogeneous TRG and HGP considering only patients with multiple lesions are reported in Table S7. No relevant pathological parameter was associated with homogenous TRG. However, in patients with multiple lesions, homogeneous HGP was associated with the absence of replacement and mixed HGP (*p* = 0.002), low homogeneous TRG (*p* = 0.018) and pathological score (*p* = 0.016).

## 4. Discussion

The choice of the best preoperative systemic treatment to improve outcome in patients with resectable CRLM is a source of debate. The BEV-ONCO trial was the first randomized study to compare preoperative administration of bevacizumab with either mFOLFOX6 or FOLFIRI in patients with resectable CRLM and evaluate pathological response as primary endpoint. Some retrospective studies already evaluated pathological response to assess the efficacy of the treatment in order to compare patients treated with chemotherapy alone (FOLFOX or FOLFIRI) and chemotherapy with anti-angiogenic treatment [14,15,16]. In particular, we previously reported that the percentage of MPRR (TRG < 3) was higher in patients who received bevacizumab with an oxaliplatin-based treatment (60% vs. 17% for irinotecan-based treatment) [16]. In the current trial, with a standardized and reproducible prospective methodology of sampling and analysis performed for all resected CRLM, we failed to demonstrate a difference in MPRR in favor of the oxaliplatin-based arm, likely due to a lack of power of the trial. Nevertheless, we observed a non-significant higher MPPR (32.1% vs. 20.7%) and complete pathological response (14.2% vs. 0%, *p* = 0.052) in patients treated with oxaliplatin-based treatment, suggesting a trend of higher efficacy of this combination. Pre-and post-operative complications were comparable in the two arms and in line with previous publications assessing surgery after bevacizumab [7,8,9,28].

As previously reported [12,13,14,15,16,17,20], our study also confirmed that TRG, HGP and pathological scores are important prognostic factors. TRG is the most widely used method to standardize pathological response evaluation [29] and is strongly associated with survival. Interestingly, we observed that more than three cycles of bevacizumab and chemotherapy were associated with worse PR and more TRG heterogeneity in resected CRLM. This would suggest that a favorable and homogenous PR could occur rapidly after the initiation of systemic treatment. HGP, especially replacement and mixed patterns, correlates with a worse prognosis after CRLM resection, as already reported [16,20].

The study explored the tumor homogeneity versus heterogeneity of the CRLM evolution after systemic treatment. Interestingly, the homogeneity of TRG and HGP observed on resected CRLM after preoperative treatment was strongly associated with PFS (HR ≤ 0.27, *p* < 0.001) and OS (HR ≤ 0.32, *p* < 0.04) but also with PR (max TRG ≤ 3: OR > 3.5, *p* ≤ 0.05). The number of CRLM per patient (1, or >1) do not completely explain this finding. Out of the 57 resected patients, 35 presented more than one lesion and, in these cases, homogeneous TRG and HGP were as equally represented as heterogeneous TRG and HGP. The biological heterogeneity of CRLM arises from different clones of cancer cells with their own genomic profile [30]. However, this evolution could be modulated by the effect of host factors and external influences such as diet, tumor immune microenvironment [21,31], cancer and gut microbiome [32,33], and systemic treatment [21,28,31]. A retrospective study [34] on 73 patients with multiple CRLM (*n* = 300) reported an association between bevacizumab treatment and homogeneous pathological response. The possible explanation was linked to the mechanism of action of this drug, inducing necrosis and modification in vasculogenesis [35]. This finding will not affect the treatment strategy but it will help to understand the tumor biology and the mechanism of the treatment.

No difference in the distribution of CALI was observed in the two arms of the study. The literature reports imply a higher prevalence of SOS and NRH in patients treated with oxaliplatin [25], but some research suggests a protective role of bevacizumab in the development of these diseases [36,37]. In our trial, while SOS prevalence was predominant (although not statistically significant) in patients treated with mFOLFOX6 (53.6% vs. 37.9% *p* = 0.501), no difference was observed between the two arms concerning the prevalence of NRH. The development of SOS was reported in around half of the patients treated with oxaliplatin alone [25]. Despite the administration of bevacizumab, the prevalence of SOS was not lower in our study. We do not find any differences in steatohepatitis occurrence in the two arms of the study. Although some articles described an association between irinotecan and steatohepatitis [38], studies involving large cohorts of patients did not report this [39,40]. These studies demonstrated that the only risk factor associated with steatohepatitis seemed to be a high patient body mass index (BMI > 27).

Our trial has several limitations. From a clinical point of view, pathological response is not a surrogate endpoint for OS and might be used in exploratory studies assessing the activity of different treatment regimens. Therefore, no definitive conclusions can be derived because of the phase two design and the activity endpoint. Moreover, the alternative hypothesis tested for the trial objective was quite ambitious. We cannot exclude an error probability in our results since the number of patients could be too small to detect an existing difference between oxaliplatin- and irinotecan-based therapies. Finally, the use of preoperative bevacizumab (and, to a lesser extent, associated chemotherapy) is not the standard of care for resectable CRLM. Nevertheless, nowadays, the definition of CRLM resectability remains highly heterogenous among surgeons. Our study could reveal relevant clinical and pathological information when a preoperative treatment is indicated before surgery.

## 5. Conclusions

To our knowledge, this is the first prospective study comparing the pathological responses of patients resected for CRLM who received mFOLFOX6 or FOLFIRI in association with bevacizumab and for whom a standardized and reproducible methodology of sampling and analysis was performed for all resected CRLM. Our study failed to demonstrate a higher MPRR or survival benefit in patients treated with mFOLFOX6-bevacizumab. Interestingly, this study highlighted the pathological response of resected CRLM as a significant biomarker for disease recurrence and revealed other pathological parameters, such as HGP, and the relation with the homogeneity of CRLM evolution as a potential prognostic marker. Even if our findings require further investigation, it certainly reinforces the need for a complete and accurate pathological evaluation of all resected CRLM, justifying a dialogue between clinicians and pathologists in clinical practice.

## Figures and Tables

**Table 1:** Clinico-pathological characteristics of the resected patient population.

CLINICAL CHARACTERISTICS		mFOLFOX+BEV *n* = 28 (100%)	FOLFIRI+BEV *n* = 29 (100%)	*p*-Value	PATHOLOGICAL CHARACTERISTICS		mFOLFOX+BEV *n* = 28 (100%)	FOLFIRI+BEV *n* = 29 (100%)	*p*-Value
**Age**	Median (iQR)	59.5 (9.5)	60.0 (13)	0.632	**Metastases number per patient (median)**	Median (iQR)	2.0 (1)	2.0 (1)	0.491
**Gender**	Female	12 (42.9%)	16 (55.2%)	0.431	**Metastases number per patient (numeric)**	1	10 (35.7%)	12 (41.4%)	0.837
	Male	16 (57.1%)	13 (44.8%)			2–3	11 (39.3%)	12 (41.4%)	
						>3	7 (25.10%)	5 (17.2%)	
**ECOG Performance Status**	PS0	18 (64.3%)	19 (65.5%)	0.999	**Metastases size per patient**	Median (iQR)	15.0 (16)	15.0 (11)	0.237
	PS	10 (35.7%)	10 (34.5%)			<20mm	18 (64.3%)	17 (58.6%)	0.787
						≥20mm	10 (35.7%)	12 (41.4%)	
**Tumor sideness**	Left	19 (67.9%)	23 (79.3%)	0.999	**Metastases resection**	R0	25 (89.3%)	27 (93.1%)	0.670
	Right	9 (32.1%)	6 (20.7%)			R1	3 (10.7%)	2 (6.9%)	
**Metastasis location**	Liver	27 (96.4%)	29 (100.0%)	0.491	**Mean TRG**	Median (iQR)	3.0 (1.4)	3.3 (1)	0.162
	Lung	1 (3.6%)	0 (0.0%)						
**Metastatic** **disease**	Synchronous	24 (85.7%)	19 (65.5%)	0.123	**Mean TRG < 3 (MPRR)**	No	19 (67.9%)	23 (79.3%)	0.379
	Metachronous	4 (14.3%)	10 (34.5%)			Yes	9 (32.1%)	6 (20.7%)	
***RAS* status**	Wild-type	8 (28.6%)	11 (37.9%)	0.576	**Max TRG ≤ 3**	No	14 (50.0%)	18 (62.1%)	0.429
	Mutated	20 (71.4%)	18 (62.1%)			Yes	14 (50.0%)	11 (37.9%)	
***BRAF* status (V600E)**	Wild-type	27 (96.4%)	29 (100.0%)	0.491	**Complete PR (TRG = 1)**	No	24 (85.7%)	29 (100.0%)	0.052
	Mutated	1 (3.6%)	0 (0.0%)			Yes	4 (14.3%)	0 (0.0%)	
**MSI/MSS**	MSS	28 (100.0%)	27 (93.1%)	0.491	**TRG homogeneous**	No	9 (32.1%)	8 (27.6%)	0.777
	MSI	0 (0.0%)	2 (6.9%)			Yes	19 (67.8%)	21 (72.4%)	
**T Stage (primary tumor)**	T1-T2	3 (10.7%)	4 (13.8%)	0.999	**TRG homogeneous low**	No	16 (57.1%)	20 (68.9%)	0.417
	T3-T4	25 (89.3%)	25 (86.2%)			Yes	12 (42.8%)	9 (31%)	
**N Stage (primary tumor)**	N0	10 (35.7%)	9 (31.0%)	0.783	**HGP dominant**	Desmoplastic	16 (57.1%)	19 (65.5%)	0.355
	N+	18 (64.3%)	20 (69.0%)			Pushing	3 (10.7%)	4 (13.8%)	
						Replacement	3 (10.7%)	0 (0.0%)	
						Mixed	3 (3 (10.7%)	5 (17.2%)	
						No dominant	1 (3.6%)	1 (3.5%)	
						NA	2 (7.1%)	0 (0.0%)	
**Number of preoperative chemotherapy cycles**	Median (iQR)	4.0 (3)	4.0 (2)	0.528	**HGP replacement and mixed**	No	13 (46.6%)	19 (65.5%)	0.190
	≤3	12 (42.9%)	7 (24.1%)	0.167		Yes	13 (46.4%)	10 (34.5%)	
	>3	16 (57.1%)	88 (75.9%)			NA	2 (7.1%)	0 (0.0%)	
**Number of preoperative BEV cycles**	Median (iQR)	3.0 (1)	3.0 (2)	0.605	**HGP homogeneous**	No	10 (35.7%)	11 (37.9%)	0.574
	≤3	20 (71.4%)	20 (69.0%)	0.999		Yes	16 (57.1%)	18 (62.1%)	
	>3	8 (28.6%)	9 (31.0%)			NA	2 (7.1%)	0 (0%)	
**Postop chemotherapy**	No	2 (7.1%)	2 (6.9%)	0.999	**Pathological Score**	0–1	21 (75.0%)	24 (82.8%)	0.530
	Yes	26 (92.9%)	27 (93.1%)			>1	7 (25.0%)	5 (17.2%)	
							
**Postop BEV**	No	17 (60.7%)	19 (65.5%)	0.787	**SOS**	No	12 (42.9%)	16 (55.2%)	0.501
	Yes	13 (39.3%)	10 (34.5%)			Yes	15 (53.6%)	11 (37.9%)	
						NA	1 (3.6%)	2 (6.9%)	
**Metastasis surgery**	One step	26 (92.8%)	27 (93.1%)	0.999	**NRH**	No	19 (67.9%)	22 (75.9%)	0.752
	Two steps	2 (7.1%)	2 (6.9%)			Yes	6 (21.4%)	5 (17.2%)	
						NA	3 (10.7%)	2 (6.9%)	
**Portal liver embolisation**	No	20 (71.4%)	21 (72.4%)	0.999	**Steatohepatitis**	No	24 (85.7%)	25 (86.2%)	0.999
	Yes	8 (28.6%)	8 (27.6%)			Yes	3 (10.7%)	4 (13.8%)	
						NA	1 (3.5%)	0 (0.0%)	

ECOG: Eastern Cooperative Oncology Group; BEV: bevacizumab; MSI: microsatellite instability; MSS: microsatellite stability; TRG: tumor regression grading; MPRR: major pathological response rate; PR: pathological response; SOS: sinusoidal obstruction syndrome; NRH: nodular regenerative hyperplasia; HGP: histopathological growth pattern; iQR: interquartile range; NA: not available; R0: negative surgical margin; R1: positive surgical margin; N0: negative lymph node; N+: positive lymph nodes.

**Table 2:** Preoperative and one-month post surgery complications.

Preoperative Complications				One-Month Post-Surgery Complications			
Characteristics	mFOLFOX + BEV *n* = 32 (100%)	FOLFIRI + BEV *n* = 32 (100%)	*p*-Value	Characteristics	mFOLFOX + BEV *n* = 28 (100%)	FOLFIRI + BEV *n* = 29 (100%)	*p*-Value
**AE grade 3–4 (all)**				**Surgical Complication**			
**Yes**	5 (15.6%)	7 (21.9%)	0.750	**No**	17 (60.7%)	20 (69.0%)	0.585
**No**	27 (84.4%)	25 (78.1%)		**Yes**	11 (39.3%)	9 (31.0%)	
**AE Special Interest**				**Single**	7 (25.0%)	4 (13.8%)	0.653
**No**	24 (75.0%)	21 (65.6%)		**Multiple**	4 (14.3%)	5 (17.2%)	
**Grade 1–2**	4 (12.5%)	5 (15.6%)		**Grade 1–2**	6 (21.4%)	7 (24.1%)	
Arterial hypertension	1	1		Wound infection	3	3	
Colon obstruction	1	0		Abdominal infection	1	4	
Pulmonary embolism	1	0		Acute renal failure	0	1	
Orthostatic syncope	0	1		Biliary leakage	0	2	
Infectious pneumonia	1	0		Venous thromboembolism	1	0	
Transient vascular cerebral ischemia	0	1		Hypovolemic shock	1	0	
Pneumothorax Skin ulcer	0 0	1 0	0.738	Upper gastrointestinal hemorrhage	0	1	0.374
**Grade 3–4**	4 (12.5%)	6 (18.7%)		**Grade 3–4**	5 (17.9%)	2 (6.9%)	
Arterial hypertension	2	4		Severe sepsis	2	1	
Lipasemia	1	0		Anastomotic leakage	2	0	
Hemorroids thrombosis	0	1		Transient liver failure	1	1	
Acute heart disfunction	1	0		Abdominal infection	4	1	
Appendicitis	0	1		Biliary leakage	2	1	
Pulmonary embolism	1	0		Thromboembolic cerebral stroke	0	1	
				Wound infection	1	0	

BEV: Bevacizumab; AE: adverse effect.

**Table 3:** Univariate logistic regression for max TRG ≤ 3.

Effect	Effect Tested	OR (CI95)	Number of Patients	*p*-Value	
Age	>65	0.994 (0.310–3.185)	57	0.992	
Gender	Male	2.598 (0.883–7.644)	57	0.083	
ECOG	PS1	0.784 (0.260–2.365)	57	0.666	
Tumor sideness	Left	1.818 (0.530–6.236)	57	0.342	
Synchronous	Yes	0.214 (0.057–0.801)	57	0.022	s
Number BEV cycles	>3	0.099 (0.020–0.490)	57	0.005	s
Number preop chemo cycles	>3	0.303 (0.096–0.956)	57	0.042	s
*RAS* status	Mutated	0.424 (0.138–1.306)	57	0.135	
Type of treatment	FOLFOX-Bev	1.636 (0.570–4.696)	57	0.360	
Lesion number	>1	0.187 (0.059–0.595)	57	0.005	s
Median lesion size	≥20	0.221 (0.066–0.733)	57	0.014	s
HGP replacement and mixed	Yes	0.190 (0.056–0.641)	55	0.007	s
HGP dominant desmoplastic	No	0.188 (0.052–0.677)	55	0.011	s
HGP homogeneous	Yes	17.416 (3.454–87.823)	55	<0.001	s
TRG homogenous	Yes	3.592 (0.998–12.932)	57	0.050	s
Pathological score	>1	0.000 (0.000–1.32E17)	57	0.950	
SOS	Yes	0.625 (0.212–1.846)	54	0.395	
NRH	Yes	1.176 (0.308–4.491)	52	0.812	
Steatohepatits	Yes	1.778 (0.359–8.808)	56	0.481	

ECOG: Eastern Cooperative Oncology Group; OR: odds ratio; TRG: tumor regression grading; HGP: histopathological growth pattern; BEV: bevacizumab; NRH: nodular regenerative hyperplasia; SOS: sinusoidal obstructive syndrome; s: significant.

**Table 4:** Univariate analysis for progression-free survival and overall survival.

		Progression-Free Survival				Overall Survival			
Effect	Effect Tested	HR	CI95	*p*-Value		HR	CI95	*p*-Value	
Age	>65	1.41	0.691–2.890	0.344		1.89	0.626–5.714	0.259	
Gender	Male	0.65	0.331–1.258	0.198		0.47	0.158–1.416	0.181	
ECOG	PS1	2.42	1.238–4.734	0.010	s	2.06	0.723–5.889	0.176	
Tumor sideness	Left	0.71	0.331–1.541	0.392		0.21	0.067–0.663	0.008	s
CEA screening category	>10	1.42	0.730–2.761	0.301		1.47	0.516–4.212	0.468	
LDH	≥250	1.66	0.829–3.307	0.153		2.47	0.861–7.089	0.093	
Synchronous/Metachronous	Yes	3.05	1.176–7.932	0.022	s	2.28	0.507–10.23	0.283	
One month surgical complication	Yes	1.58	0.809–3.101	0.179		1.89	0.662–5.390	0.235	
*RAS* status	Mutated	0.83	0.417–1.644	0.589		1.28	0.403–4.096	0.673	
Type of treatment	mFOLFOX6-BEV	1.18	0.607–2.291	0.626		1.38	0.479–4.003	0.550	
Lesion number	>1 lesion	2.38	1.135–4.982	0.022	s	1.96	0.600–6.405	0.265	
Median lesion size	≥20	1.88	0.961–3.678	0.065		2.14	0.743–6.165	0.158	
Status of the margin	R1	3.57	1.215–10.48	0.021	s	1 847	0.409–8.338	0.425	
Pathological complete response	Yes	0.66	0.159–2.777	0.576		0.90	0.117–6.921	0.918	
Max TRG ≤ 3	Yes	0.41	0.202–0.835	0.014	s	0.34	0.105–1.114	0.075	
Mean TRG < 3	Yes	1.20	0.575–2.505	0.628		1.49	0.497–4.461	0.477	
TRG homogeneus	Yes	0.21	0.101–0.435	<0.001	s	0.23	0.073–0.701	0.010	s
TRG homogeneous low	Yes	0.33	0.151–0.712	0.005	s	0.30	0.081–1.097	0.069	
HGP homogenous	Yes	0.27	0.137–0.543	<0.001	s	0.32	0.107–0.932	0.037	s
HGP dominant desmoplastic	No	1.71	0.873–3.368	0.118		0.61	0.190–1.955	0.405	
HGP replacement and mixed	Yes	2.21	1.121–4.375	0.022	s	1.24	0.426–3.586	0.697	
Pathological score	>1	2.46	1.172–5.155	0.017	s	2.24	0.680–7.379	0.185	
SOS	Yes	1.28	0.642–2.570	0.480		2.76	0.815–9.355	0.103	
NRH	Yes	0.57	0.218–1.481	0.248		1.39	0.375–5.162	0.622	
Steatohepatitis	Yes	0.73	0.256–2.090	0.559		0.00	0.00–NE	0.994	

ECOG: Eastern Cooperative Oncology Group; HR: Hazard ratio; CI: confidence interval; TRG: tumor regression grading; HGP: histopathological growth pattern; BEV: bevacizumab; R1: positive resection margin; CEA: carcinoembryonic antigen; LDH: lactate dehydrogenase; NRH: nodular regenerative hyperplasia; SOS: sinusoidal obstructive syndrome; NE: non estimated; s: significant.

## Data Availability

For data supporting the results of this study, contact the corresponding author.

## Supplementary Materials

The following are available online at https://www.mdpi.com/article/10.3390/cancers14051183/s1, Supplementary Materials and Methods, Figure S1: Study design, Figure S2: Pathological parameters evaluated, Figure S3: Impact on PFS and OS of homogeneous TRG and HGP, Table S1: Clinical characteristics of the patient population included in the study, Table S2: Pathological characteristics of CRLM, Table S3: Univariated logistic regression for homogeneous TRG and homogeneous HGP, Table S4. Comparison between one and multiple lesions group, Table S5 Univariated logistic regression for homogeneous TRG and homogeneous HGP in multiple lesions. Table S6. Comparison between one and multiple lesions group, Table S7. Univariate logistic regressions for homogeneous TRG and homogeneous HGP in multiple lesions.
