# Supplementary material for: Randomized Phase 2 Study Comparing Pathological Responses of Resected Colorectal Cancer Metastases after Bevacizumab with mFOLFOX6 or FOLFIRI (BEV-ONCO Trial)
Source: Cancers (Basel). 2022 Feb 24;14(5):1183. doi: 10.3390/cancers14051183 (PMC8909786; doi:10.3390/cancers14051183)
Supplement: Supplementary file 1 [file cancers-14-01183-s001.zip › cancers-1555639-supplementary.pdf]

cancers

MDPI

Supplementary Materials

Randomized Phase 2 Study Comparing Pathological Responses of Resected Colorectal Cancer Metastases After Bevacizumab with mFOLFOX6 or FOLFIRI (BEV-ONCO Trial)

Pamela Baldin [1,†], Javier Carrasco [2,†], Gabriela Beniuga [3], Anne Jouret-Mourin [1,3], Gauthier Demolin [4], Sandrine Roland [5], Lionel D'Hondt [6], Philippe Vergauwe [7], Daniel Van Daele [8], Marie Mailleux [9], Isabelle Sinapi [2], Astrid De Cuyper [10], Noëlla Blétard [11], Brigitte Massart [11], Monique Delos [12], Marie-Laure Castella [13], Aline van Maanen [14] and Marc Van den Eynde [15,*]

1 Pathology Department, Cliniques Universitaires Saint Luc (UCL)—Université Catholique de Louvain, 1200 Bruxelles, Belgium; pamela.baldin@uclouvain.be (P.B.); anne.mourin@uclouvain.be (A.J.-M.)
2 Medical Oncology Department, GHdC-Grad Hopital de Charleroi-Site Notre Dame, 6000 Charleroi, Belgium; javier.carrasco@ghdc.be (J.C.); isabelle.sinapi@ghdc.be (I.S.)
3 Pathology Department, Institut de Pathologie et Génétique, 6041 Gosselies, Belgium; gabriela.beniuga@ipg.be
4 Gastroenterology Department, Clinique CHC MonLégia, 4000 Liège, Belgium; gauthier.demolin@chc.be
Gastroenterology Department, CHIREC-Hôpital Delta, 1160 Auderghem, Belgium; sandrine.roland@chirec.be
6 Oncology Department, CHU-UCL-Namur, Site Godinne, 5530 Yvoir, Belgium; lionel.dhondt@uclouvain.be
7 Gastroenterology Department, AZ Groeninge Hospital, 3220 Kortrijk, Belgium; philippe.vergauwe@azgroeninge.be
8 Gastroenterology Department, CHU de Liège, 4000 Liège, Belgium; daniel.vandaele@chu.ulg.ac.be
9 Medical Oncology, Clinique Saint-Luc Bouge, 5000 Namur, Belgium; marie.mailleux@slbo.be
10 Department of Medical Oncology, Cliniques Universitaires Saint Luc (UCL)—Université Catholique de Louvain, 1200 Bruxelles, Belgium; astrid.decuyper@uclouvain.be
11 Pathology Department, Clinique CHC MonLégia, 4000 Liège, Belgium; noella.bletard@chc.be (N.B.); brigitte.massart@chc.be (B.M.)
12 Pathology Department, CHU-UCL-Namur, Site Godinne, 5530 Yvoir, Belgium; monique.delos@uclouvain.be
13 Colorectal Clinical Research Unit, Institut Roi Albert II, Cliniques Universitaires Saint Luc (UCL)—Université Catholique de Louvain, 1200 Bruxelles, Belgium; marie-laure.castella@uclouvain.be
14 Support Statistique, Institut Roi Albert II, Cliniques Universitaires Saint Luc (UCL)—Université Catholique de Louvain, 1200 Bruxelles, Belgium; aline.vanmaanen@uclouvain.be
15 Department of Medical Oncology and Gastroenterology, Cliniques Universitaires Saint Luc (UCL)—Université Catholique de Louvain, 1200 Bruxelles, Belgium
* Correspondence: marc.vandeneynde@uclouvain.be
† These authors contributed equally to this paper.

Supplemental Materials and Methods

1. Study Design and Patients

Included patients were randomized by a blocked stratified central randomization in two arms with a ratio of 1:1.

Regarding eligibility criteria, an extrahepatic metastatic location limited to one site and easily resectable was allowed. Prior limited CRLM resection was allowed within 3 months before inclusion if patient never received systemic therapy for mCRC. Prior adjuvant chemotherapy was permitted if it was completed more than 6 months prior to inclusion (12 months if oxaliplatin-based chemotherapy) and without residual toxicity (polyneuropathy < grade 2).

Key exclusion criteria included non resectable CRLM; prior utilization of anti-VEGF therapy and any clinical situations which could compromise CRLM surgery or systemic treatment administration (ascites, cirrhosis or portal hypertension; prior major liver resection).

The study was conducted in 8 hospitals in Belgium listed thereafter: Cliniques universitaires St-Luc (Brussels), Grand Hôpital de Charleroi, Clinique CHC du Montlégia (Liège), CHU de Liège, CHU—UCL Namur, Hôpital Delta – CHIREC (Brussels), AZ Groeninge Hopistal (Kortrijk) and Clinique Saint-Luc (Bouge).

Patient received up to 6 cycles of preoperative treatment. On day 1 of each 14-days treatment cycle, patients received bevacizumab (5mg/kg 90 min infusion for 1st cycle, 60 min for the 2nd and 30 min from the 3rd cycle), and mFOLFOX6 (arm A), comprising 2 hours synchronous infusion of oxaliplatin 85 mg/m2 and levoleucovorin 200mg/m2 (or folinic acid 400mg/m2), a bolus of 5-FU 400mg/m2 and then a continuous 46 hours infusion of 5-FU 2400 mg/m2 or FOLFIRI (arm B: 90 min infusion of irinotecan 180mg/m2 and 2 hours levoleucovorin 200mg/m2 (or folinic acid 400mg/m2), a bolus of 5-FU 400mg/m2 and then a continuous 46 hours infusion of 5-FU 2400 mg/m2). Patients were operated within 4 to 8 weeks after the last chemotherapy cycle and following local and institutional procedure.

2. Pathological Evaluation

A protocol with the guidelines for the handling of the surgical specimens has been proposed to the different participating centers, in order to make the sampling homogeneous.

The surgical specimens were cut in 5-mm thick slices. In patients with multiple liver metastases, each lesion was measured and sampled for analysis. If the lesion was smaller than 25 mm, it was completely sampled. If the lesion was bigger than 25mm and cannot be completely sampled, one complete slice on the center of the lesion was collected. Moreover, additional samples were taken in the residual tissue perpendicular to the first slice in order to sample at least the two major axis of the lesion. For each patient, we collected a piece from the surrounding non tumoral liver parenchyma, far from the CRLM, in order to evaluate the presence of chemotherapy associated liver injury (CALI). In addition, according to sufficient tumor supply, a specimen for each patient from the three most representative metastases and a specimen from the surrounding parenchyma have been frozen and stored at −80 °C in the tumor-bank.

The same procedure described for CRLM was applied for extrahepatic metastases when possible.

3. Objectives, Statistical Considerations and Analyses

3.1. One-Month Surgical Complication Rate Is Defined:

1. Severe pre- or postoperative complications within 30 days of surgery:
    - surgery-associated bleeding requiring replacement with >4 units of erythrocyte concentrates,
    - wound infection,
    - intra-abdominal infection,
    - severe sepsis (American College of Chest physicians/Society of Critical Care Medicine, 1992),
    - impaired wound healing,
    - subphrenic or perihepatic abscess requiring drainage during hospital stay or within 30 days after the operation,
    - re-laparotomy connected with the resection,
    - a biliary fistula for more than 10 days with a discharge of >100 mL/day,
    - transient liver failure (bilirubin >10 mg/dL lasting >3 days),
    - renal failure requiring dialysis,
    - respiratory failure with renewed necessary mechanical ventilation, venous thromboembolism,
    - cardiac failure,
    - death of the patient as a result of the operation.

2. Any other severe operative or post-operative complication requiring prolongation of hospitalization, re-operation (excluding secondary surgery after R1 resection), or resulting in death, within 30 days of surgery.

Toxicity is evaluated according to the Common Terminology Criteria for Adverse Events criteria (CTCAE version 4.0). Late and persistent toxicities will continue to be followed as required.

A sample size of 54 patients (27 per arm) was needed to achieve 80% power to detect a difference between the group proportions of 0.40 for MPPR. The proportion in the treatment group FOLFIRI + bevacizumab is assumed to be 0.20 under the null hypothesis and 0.60 under the alternative hypothesis. The proportion in the treatment group FOLFIRI + bevacizumab is 0.20. Type I error was set as 0.05. With an expected drop-out rate of 10%, 60 subjects will be randomized. The statistic test used for MPPR comparison between arms is the two-sided Fisher's Exact test.

Continuous variables were summarized as median and interquartile range (IQR) and group comparisons were analyzed using a Mann-Whitney U-test. Categorical variables were summarized as numbers and percentage, and comparisons performed using a Fisher's Exact test.

Univariate logistic regression modelling was used to identify factors affecting TRG and HGP.

PFS is defined as the time from randomization to the relapse or death and OS as time from randomization to death of any cause. Patients with no event (relapse or death as appropriate) at the time of analysis were censored at the date of last follow-up. Patients with no follow-up after randomization were censored on day 1 (date of randomization).

PFS and OS were summarized using Kaplan–Meier curves. Univariate then multivariate Cox Proportional Hazard modelling was used to identify survival risk factors, providing hazard ratios (HRs), 95% confidence intervals (CIs) and Wald's *p*-values. Backward stepwise selection was used to select optimal multivariate model for OS and PFS. Potential collinearity was tested among the multiple parameters significantly associated with survival using the variance indicator factor (VIF) and the collinearity indeces (COLLIN). All *p*-values were 2-tailed, and *p*-values of less than 0.05 were considered statistically significant. Analysis was performed using SAS software (Version 9.4; SAS Institute Inc, Cary, NC, USA).

Supplemental Figures and Tables

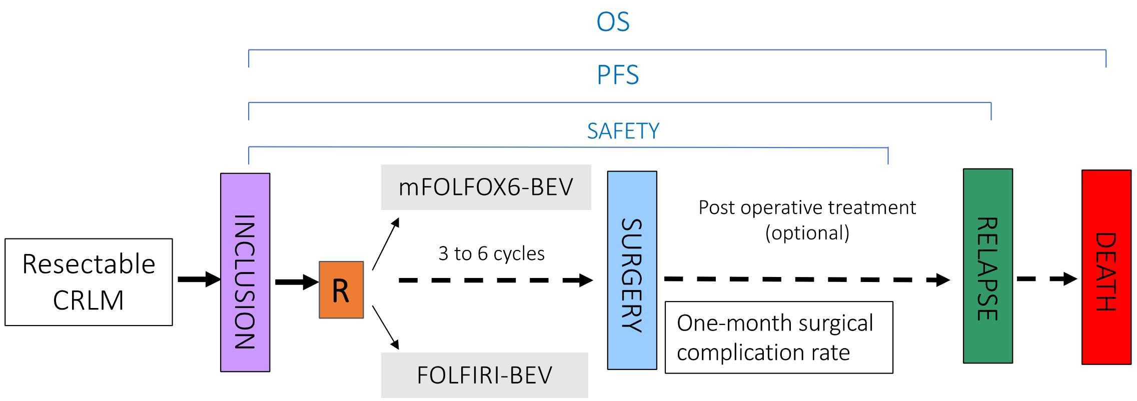

Figure S1. Study design. BEV: Bevacizumab, CRLM: colorectal liver metastases, PFS: progression-free survival, R: randomization, OS: Overall survival.

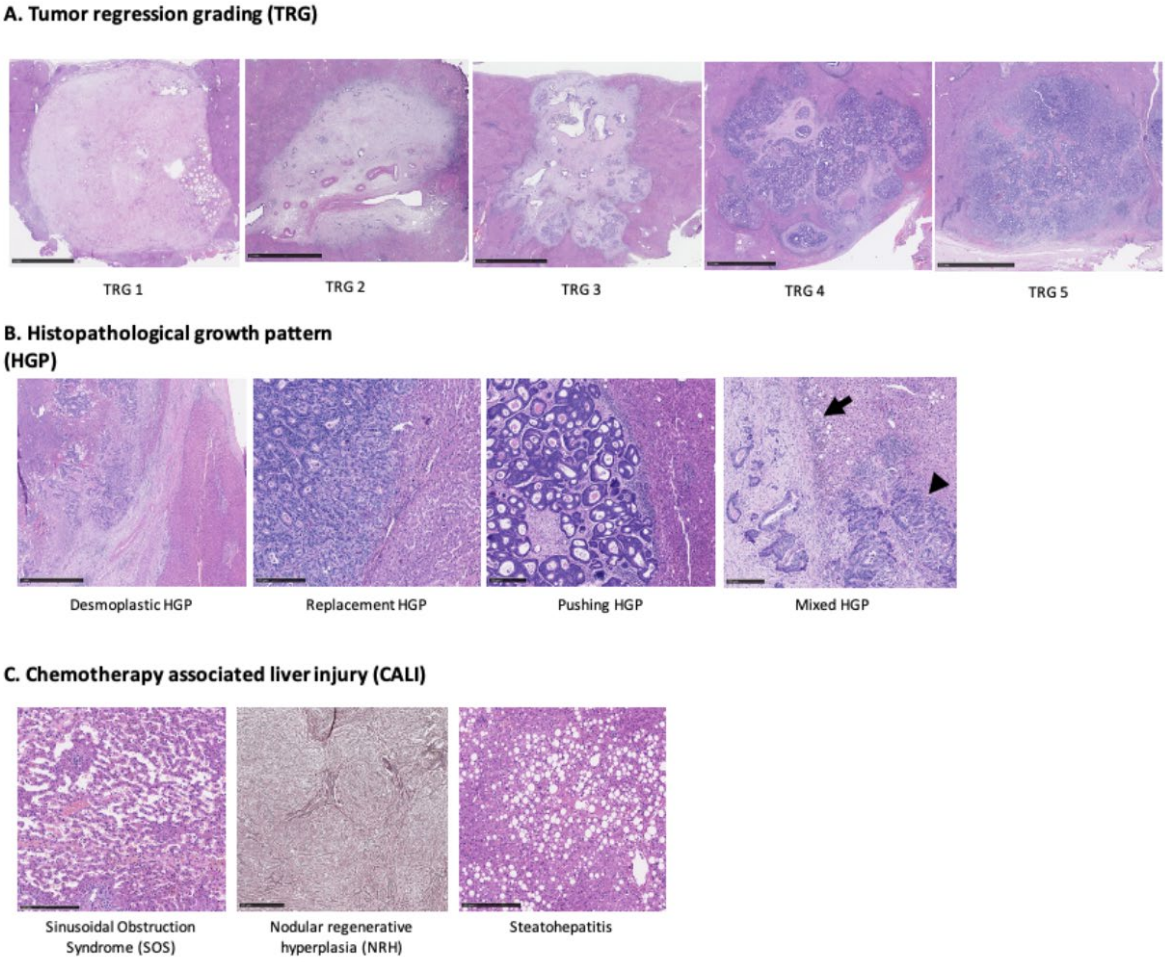

Figure S2. Pathological parameters evaluated. (**A**) TRG in colorectal liver metastases (H&E). TRG 1 (complete response, 0.55× magnification); TRG 2 (only scattered neoplastic cells, 1.25× magnification); TRG 3 (more residual tumor cells but fibrosis predominates 1.25× magnification); TRG 4 (residual cancer cells predominating over fibrosis, 1.25× magnification); and TRG 5 (no signs of regression 1.25× magnification). (**B**) HGPs (H&E). Desmoplastic HGP (metastasis surrounded by a desmoplastic rim, 1.25× magnification), pushing HGP (metastasis grows by compressing the liver parenchyma, 5× magnification), replacement HGP (metastases growth preserves the architecture of the hepatic tissue, 7× magnification) and mixed HGP (a mix of two or more patterns, desmoplastic HGP (arrow) and replacement HGP (arrowhead) are shown here, 7× magnification). (**C**) Chemotherapy related liver injury (CALI) is classified in sinusoidal obstructive syndrome (SOS, H&E, 10× magnification) (vascular- endothelial damage); NRH (reticulin staining, 10× magnification) (nodularity aspect of the liver parenchyma without fibrosis); and steatohepatitis (H&E, 10× magnification).

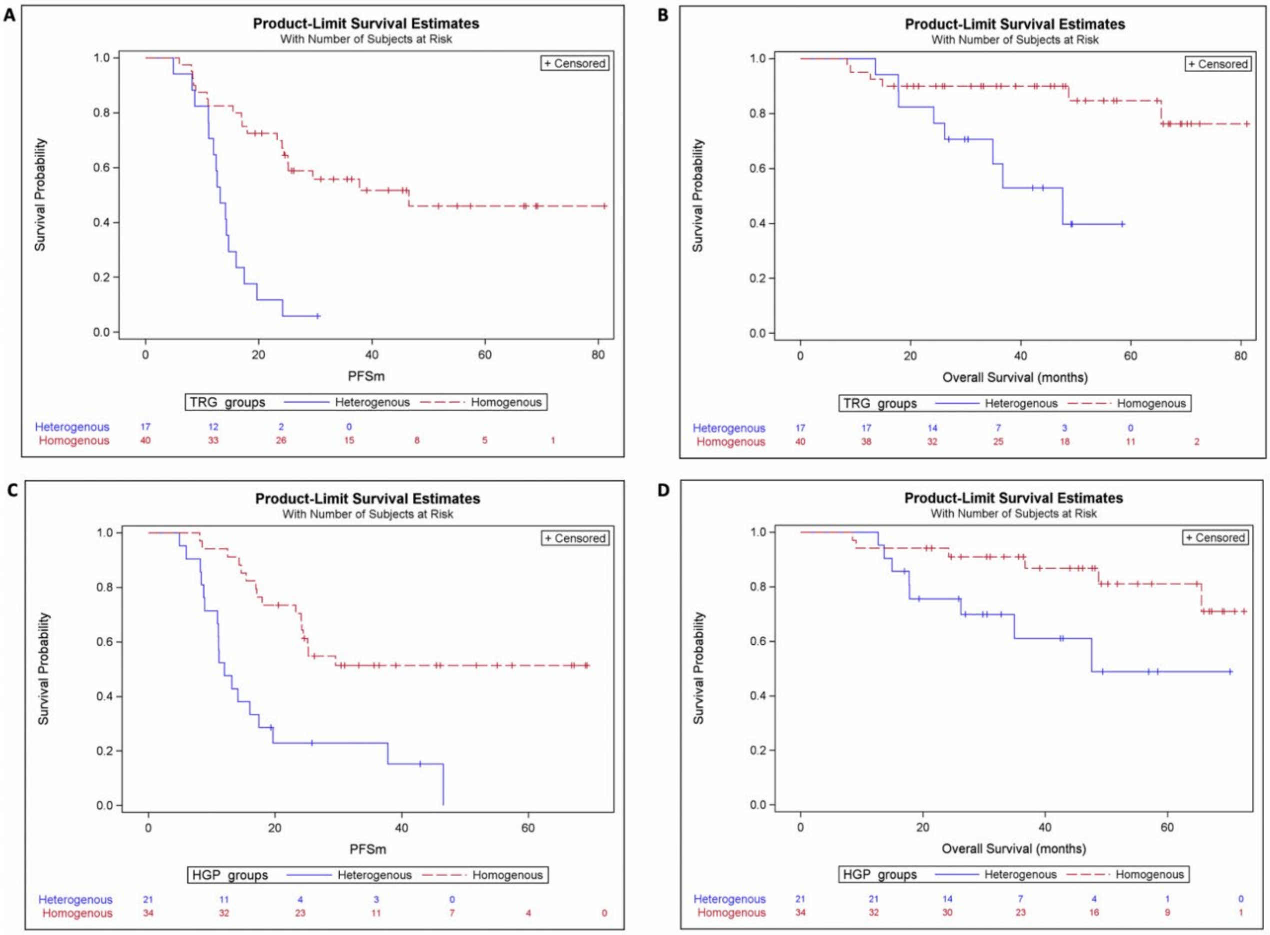

Figure S3. Impact on PFS and OS of homogeneous TRG and HGP. Kaplan-Meyer curves for PFS (**A**) and OS (**B**) according to homogeneous or heterogeneous TRG. Kaplan-Meyers curves for PFS (**C**) ads OS (**D**) of homo- and heterogeneous HGP.

Table S1. Clinical characteristics of the patient population included in the study.

**Clinical Characteristics**		**mFOLFOX6—BEV** *n* = 33	**FOLFIRI—BEV** *n* = 32	***p*-value**
**Age**	Median (IQR)	60.0 (10.2)	62.0 (9.4)	0.599
**Gender**	Female	13 (39.4%)	18 (56.2%)	0.218
	Male	20 (60.6%)	14 (43.7%)	
**ECOG Performance Status**	PS0	22 (66.8%)	22 (68.7%)	0.999
	PS1	11 (33.3%)	10 (31.3%)	
**Tumor sideness**	Left	22 (66.7%)	26 (81.2%)	0.999
	Right	1 (33.3%)	6 (18.7%)	
**Metastasis location**	Liver	32 (96.9%)	32 (100.0%)	0.999
	Lung	1 (3.1%)	0 (0.0%)	
**Metastatic disease**	Synchronous	29 (87.9%)	21 (65.6%)	0.042
	Metachronous	4 (12.1%)	11 (34.4%)	
***RAS* status**	Wild-type	10 (30.3%)	13 (40.6%)	0.443
	Mutated	23 (69.7%)	19 (59.4%)	
***BRAF* status (V600E)**	Wild-type	30 (90.9%)	30 (96.9%)	1.000
	Mutated	1 (3.0%)	0 (0.0%)	
	Unknown	2 (6.1%)	1 (3.1%)	
**MSI/MSS status**	MSS	30 (90.9%)	28 (87.5%)	0.833

	MSI	0 (0.0%)	1 (3.1)	
	Unknown	3 (9.1%)	3 (9.4%)	
**T Stage (primary tumor)**	T1-T2	3 (9.1%)	4 (12.5%)	0.166
	T3	19 (57.6%)	24 (75.0%)	
	T4	11 (33.3%)	4 (12.5%)	
**N stage (primary tumor)**	N0	13 (39.4%)	11 (34.4%)	0.798
	N+	20 (66.6%)	21 (65.6%)	

BEV: bevacizumab; MSI: microsatellite instability; MSS: microsatellite stability; iQR: interquartile range.

Table S2. Pathological characteristics of CRLM.

Pathological characteristics		mFOLFOX6-BEV *n* = 89	FOLFIRI-BEV *n* = 70	*p*-value
**Lesion size (mm)**	median (IQR)	10 (16)	17 (13)	0.002
**Status of the margin**	R0	87 (97.7%)	67 (95.7%)	
	R1	2 (2.2%)	3 (4.3%)	0.655
**TRG**	TRG ≤3	47 (52.8%)	30 (42.86%)	
	TRG 4–5	42 (47.2%)	40 (57.1%)	0.263
**HGP**	Desmoplastic	45 (50.6%)	35 (50%)	
	Replacement	11 (12.4%)	4 (5.7%)	
	Pushing	15 (16.8%)	14 (20%)	
	Mixed	13 (14.6%)	17 (24.3%)	
	NA	5 (5.6%)	0 (0%)	0.096

CRLM: colorectal liver metastasis; BEV: bevacizumab; IQR: interquartile range; R0: negative surgical margin, R1: positive surgical margin; TRG: tumor regression grading; HGP: histopathological growth pattern, NA: non available.

Table S3. Univariate logistic regression for homogeneous TRG and homogeneous HGP.

		Homogeneous TRG				Homogeneous HGP			
**Effect**	**Effect Tested**	**OR (CI95)**	***n***	***p*-Value**		**OR (CI95)**	***n***	***p*-Value**	
Age	>65	1.393 (0.376–5.156)	57	0.620		1.680 (0.505–5.581)	57	0.397	
Gender	Male	1.243 (0.399–3.875)	57	0.707		2.833 (0.952–8.434)	57	0.061	
ECOG	PS1	0.482 (0.150–1.550)	57	0.221		0.394 (0.130–1.198)	57	0.101	
Tumor sideness	Left	0.811 (0.217–3.031)	57	0.756		1.061 (0.324–3.475)	57	0.922	
Synchronous	Yes	0.000 (0.000–3.22E18	57	0.953		0.211 (0.049–0.912)	57	0.037	s
Number BEV cycles	>3	0.222 (0.065–0.759)	57	0.016	s	0.437 (0.139–1.375)	57	0.157	
Number preop chemo cycles	>3	0.180 (0.036–0.896)	57	0.036	s	0.617 (0.198–1.922)	57	0.405	
RAS mutated	Mutated	0.774 (0.226–2.645)	57	0.683		1.548 (0.512–4.683)	57	0.439	
Type of treatment	mFOLFOX6-BEV	0.804 (0.258–2.506)	57	0.707		1.312 (0.459–3.755)	57	0.612	
Lesion number	>1	0.000 (0.000–1.94E14)	57	0.940		0.242 (0.072–0.807)	57	0.021	s
Median lesion size (mm)	≥20	0.857 (0.269–2.731)	57	0.7943		0.446 (0.150–1.321)	57	0.145	
HGP replacement and mixed	Yes	0.300 (0.089–1.008)	55	0.051		0.029 (0.006–0.135)	55	<0.001	s
HGP dominant desmoplastic	No	1.375 (0.399–4.744)	55	0.614		0.160 (0.048–0.536)	55	0.003	s
HGP homogeneous	Yes	6.380 (1.777–22.907)	55	0.004	s	/	/	/	
TRG homogeneous	Yes	/	/	/		4.687 (1.411–15.564)	57	0.012	s
TRG homogenous Low	Yes	/	/	/		13.569 (2.708–68.003)	57	0.002	s
Pathoscore	>1	0.324 (0.086–1.212)	57	0.094		0.026 (0.003–0.226)	57	<0.001	s
SOS	Yes	0.267 (0.071–0.999)	54	0.050	s	0.317 (0.101–0.991)	54	0.048	s
NRH	Yes	0.978 (0.219–4.364)	52	0.976		3.522 (0.675–18.367)	54	0.135	
Steatohepatits	Yes	2.647 (0.293–23.947)	56	0.386		1.147 (0.224–5.862)	56	0.869	

TRG: tumor regression grading; HGP: histopathological growth pattern; OR: odds ratio; *n*: number of patients; BEV: bevacizumab; NRH: nodular regenerative hyperplasia; SOS: sinusoidal obstructive syndrome; s: significant.

Table S4. Collinearity analysis for factors associated with PFS and OS.

Collinearity Diagnostics (Intercept Adjusted)							
			Proportion of Variation				
Number	Eigenvalue	Condition Index	Max TRG ≤3	TRG homogeneous	HGP homogeneous	Synchronous	Lesion number >1
**1**	2.66593	1.00000	0.04292	0.04560	0.04819	0.04876	0.04950
**2**	0.85631	1.76444	0.33295	0.16502	0.13165	0.06770	0.05603
**3**	0.64168	2.03828	0.01175	0.44522	0.08350	0.47684	0.01681
**4**	0.47023	2.38106	0.28482	0.00066697	0.45237	0.24775	0.31850
**5**	0.36585	2.69944	0.32755	0.34350	0.28428	0.15895	0.55917
**Parameter Estimates and VIF for OS dependant factor**							
**Variable**	**DF**	**Parameter Estimate**	**Standard Error**	**t Value**	**Pr > |t|**	**Tolerance**	**Variance Inflation**
**Intercept**	**1**	27.34212	9.35723	2.92	0.0052		0
**Max TRG ≤3**	**1**	9.86951	5.90554	1.67	0.1011	0.66084	1.51323
**TRG homogeneous**	**1**	5.41038	6.38258	0.85	0.4007	0.67455	1.48248
**HGP homogeneous**	**1**	5.54630	6.26672	0.89	0.3805	0.61152	1.63527
**Synchronous**	**1**	1.56280	6.68430	0.23	0.8161	0.66860	1.49567
**Lesion number >1**	**1**	0.46562	6.58273	0.07	0.9439	0.54506	1.83468
**Parameter Estimates and VIF for PFS dependant factor**							
**Variable**	**DF**	**Parameter Estimate**	**Standard Error**	**t Value**	**Pr > |t|**	**Tolerance**	**Variance Inflation**
**Intercept**	**1**	17.32672	8.05276	2.15	0.0364		0
**Max TRG ≤3**	**1**	5.94997	5.08227	1.17	0.2474	0.66084	1.51323
**TRG homogeneous**	**1**	10.79241	5.49280	1.96	0.0551	0.67455	1.48248
**HGP homogeneous**	**1**	6.39725	5.39309	1.19	0.2413	0.61152	1.63527
**Synchronous**	**1**	-8.32999	5.75246	-1.45	0.1540	0.66860	1.49567
**Lesion number >1**	**1**	2.04788	5.66505	0.36	0.7193	0.54506	1.83468

Table S5. Uni and Multivariate analysis for PFS and OS.

Univariate and Multivariate Analysis for Overall Survival							
		Univariate Analysis			Multivariate Analysis		
**Effect**	**Effect Tested**	**HR (CI95)**	***p*-Value**		**HR**	***p*-Value**	
Synchronous	Yes	2.28 (0.507–10.23)	0.283				
Lesion number	>1	1.96 (0.600–6.405)	0.265				
Max TRG ≤3	Yes	0.34 (0.105–1.114)	0.075				
TRG homogeneous	Yes	0.23 (0.073–0.701)	0.010	s	0.22 (0.071–0.677)	0.008	s
HGP homogeneous	Yes	0.32 (0.107–0.932)	0.037	s			
**Univariate and Multivariate analysis for Progression-Free Survival**							
		**Univariate analysis**			**Multivariate analysis**		
**Effect**	**Effect tested**	**HR (CI95)**	***p*-value**		**HR**	***p*-value**	
Synchronous	Yes	3.05 (1.176–7.932)	0.022	s			
Lesion number	>1	2.38 (1.135–4.982)	0.022	s			
Max TRG ≤3	Yes	0.41 (0.202–0.835)	0.014	s			
TRG homogeneous	Yes	0.21 (0.101–0.435)	<0.001	s	0.30 (0.135–0.664)	0.003	s
HGP homogeneous	Yes	0.27 (0.137–0.543)	<0.001	s	0.37 (0.176–0.768)	0.008	s

HR: hazard ratio; TRG: tumor regression grading; HGP: histopathological growth pattern; s: significant.

Table S6. Comparison between one and multiple lesions group.

		**Single Lesion** n **= 22**	**Multiple Lesions** n **= 35**	**Total** n **= 57**	***p*-Value**
TRG	Heterogeneous	0 (0%)	17 (48.57%)	17 (29.82%)	
	Homogeneous	22 (100%)	18 (51.43%)	40 (70.18%)	<0.001

HGP	Heterogeneous	3 (13.64%)	18 (51.43%)	21 (36.84%)	
	Homogeneous	19 (86.36%)	15 (42.86%)	34 (59.65%)	
	NA	0 (0%)	2 (5.71%)	2 (3.51%)	0.004
HGP/TRG	Heterogeneous	3 (13.64%)	23 (65.71%)	26 (45.61%)	
	Homogeneous	19 (86.36%)	10 (28.57%)	29 (50.88%)	
	NA	0 (0%)	2 (5.71%)	2 (3.51%)	<0.001

N: number of patients; TRG: tumor regression grading; HGP: histopathological growth pattern; NA: non available.

Table S7. Univariate logistic regressions for homogeneous TRG and homogeneous HGP in multiple lesions.

		Homogeneous TRG			Homogeneous HGP			
**Effect**	**Effect Tested**	**OR (CI95)**	***n***	***p*-Value**	**OR (CI95)**	***n***	***p*-Value**	
Age	>65	1.625 (0.367–7.201)	35	0.523	2.429 (0.568–10.389)	35	0.231	
Gender	Male	1.125 (0.298–4.240)	35	0.862	3.239 (0.834–12.578)	35	0.090	
ECOG	PS1	0.433 (0.106–1.761)	35	0.242	0.287 (0.068–1.217)	35	0.090	
Tumor sideness	Left	0.615 (0.139–2.727)	35	0.523	1.401 (0.327–5.993)	35	0.649	
Synchronous	Yes	0.000 (0.000–3.93E26)	35	0.969	0.205 (0.011–3.774)	35	0.286	
Number BEV cycles	>3	0.444 (0.113–1.743)	35	0.245	0.595 (0.158–2.244)	35	0.443	
Number preop chemo cycles	>3	0.210 (0.036–1.210)	35	0.081	0.829 (0.189–3.631)	35	0.804	
RAS status	Mutated	0.833 (0.199–3.487)	35	0.803	2.250 (0.524–9.665)	35	0.276	
Type of treatment	mFOLFOX6-BEV	0.889 (0.236–3.351)	35	0.862	1.367 (0.373–5.018)	35	0.637	
Median lesion size	≥20	1.429 (0.375–5.437)	35	0.601	0.738 (0.200–2.726)	35	0.649	
HGP replacement and mixed	Yes	0.675 (0.168–2.709)	33	0.579	0.073 (0.013–0.393)	33	0.002	s
HGP dominant desmoplastic	No	2.475 (0.597–10.268)	33	0.212	0.291 (0.067–1.271)	33	0.101	
HGP homogeneous	Yes	3.142 (0.751–13.157)	33	0.117	/	/	/	
TRG homogeneous	Yes	/	/	/	2.586 (0.678–9.872)	35	0.164	
TRG homogenous low	Yes	/	/	/	18.087 (1.640–199.43)	35	0.018	s
Pathological score	>1	0.705 (0.168–2.955)	35	0.633	0.049 (0.005–0.458)	35	0.008	s
SOS	Yes	0.255 (0.057–1.138)	32	0.073	0.337 (0.081–1.399)	32	0.134	
NRH	Yes	0.786 (0.132–4.680)	31	0.791	3.000 (0.459–19.591)	31	0.251	
Steatohepatitis	Yes	4.286 (0.426–43.139)	34	0.217	2.984 (0.424-–20.993)	34	0.272	

TRG: tumor regression grading; HGP: histopathological growth pattern; BEV: bevacizumab; OR: odds ratio; N: number of patients; NRH: nodular regenerative hyperplasia; SOS: sinusoidal obstructive syndrome; s: significant.
